# The *cwp66* Gene Affects Cell Adhesion, Stress Tolerance, and Antibiotic Resistance in Clostridioides difficile

**DOI:** 10.1128/spectrum.02704-21

**Published:** 2022-03-31

**Authors:** Qingshuai Zhou, Fengqin Rao, Zhenghong Chen, Yumei Cheng, Qifang Zhang, Jie Zhang, Zhizhong Guan, Yan He, Wenfeng Yu, Guzhen Cui, Xiaolan Qi, Wei Hong

**Affiliations:** a Key Laboratory of Endemic and Ethnic Diseases, Ministry of Education & Key Laboratory of Medical Molecular Biology of Guizhou Province, Guizhou Medical University, Guiyang, Guizhou, China; b Key Laboratory of Microbiology and Parasitology of Education Department of Guizhou, School of Basic Medical Science, Guizhou Medical University, Guiyang, Guizhou, China; c Department of Critical Care Medicine, the Affiliated Hospital of Guizhou Medical University, Guiyang, Guizhou, China; d State Key Laboratory of Animal Nutrition, Institute of Animal Science, Chinese Academy of Agricultural Sciences, Beijing, China; University of Guelph

**Keywords:** *Clostridioides difficile*, CRISPR-Cpf1, cell wall protein 66 (Cwp66), phenotypic analysis, transcriptome analysis

## Abstract

Clostridioides difficile is a Gram-positive, spore-forming anaerobic bacteria that is one of the leading causes of antibiotic-associated diarrhea. The cell wall protein 66 gene (*cwp66*) encodes a cell wall protein, which is the second major cell surface antigen of C. difficile. Although immunological approaches, such as antibodies and purified recombinant proteins, have been implemented to study the role of Cwp66 in cell adhesion, no deletion mutant of the *cwp66* gene has yet been characterized. We constructed a *cwp66* gene deletion mutant using Clustered Regularly Interspaced Short Palindromic Repeats Cpf1 (CRISPR-Cpf1) system. The phenotypic and transcriptomic changes of the Δ*cwp66* mutant compared with the wild-type (WT) strain were studied. The deletion of the *cwp66* gene led to the decrease of cell adhesive capacity, cell motility, and stresses tolerance (to Triton X-100, acidic environment, and oxidative stress). Interestingly, the Δ*cwp66* mutant is more sensitive than the WT strain to clindamycin, ampicillin, and erythromycin but more resistant than the latter to vancomycin and metronidazole. Moreover, mannitol utilization capability in the Δ*cwp66* mutant was lost. Comparative transcriptomic analyses indicated that (i) 22.90-fold upregulation of *cwpV* gene and unable to express *gpr* gene were prominent in the Δ*cwp66* mutant; (ii) the *cwp66* gene was involved in vancomycin resistance of C. difficile by influencing the expression of d-Alanine-d-Alanine ligase; and (iii) the mannose/fructose/sorbose IIC and IID components were upregulated in Δ*cwp66* mutant. The present work deepens our understanding of the contribution of the *cwp66* gene to cell adhesion, stress tolerance, antibiotic resistance, and mannitol transportation of C. difficile.

**IMPORTANCE** The cell wall protein 66 gene (*cwp66*) encodes a cell wall protein, which is the second major cell surface antigen of C. difficile. Although immunological approaches, such as antibodies and purified recombinant proteins, have been implemented to study the role of Cwp66 in cell adhesion, no deletion mutant of the *cwp66* gene has yet been characterized. The current study provides direct evidence that the *cwp66* gene serves as a major adhesion in C. difficile, and also suggested that deletion of the *cwp66* gene led to the decrease of cell adhesive capacity, cell motility, and stresses tolerance (to Triton X-100, acidic environment, and oxidative stress). Interestingly, the antibiotic resistance and carbon source utilization profiles of the Δ*cwp66* mutant were significantly changed. These phenotypes were detrimental to the survival and pathogenesis of C. difficile in the human gut and may shed light on preventing C. difficile infection.

## INTRODUCTION

Clostridioides difficile (also known as Clostridium difficile) is a Gram-positive, end-spore-forming, strict anaerobe (1). It has become one of the leading causes of nosocomial antibiotic-associated diarrheas (ADD) worldwide. About 15% of all hospitalized patients who received antibiotic treatment developed AAD, with nearly 20% to 30% of AAD caused by C. difficile (2). Therefore, research on the pathogenesis of C. difficile has attracted extensive attention worldwide (3).

After ingestion of C. difficile spores or vegetative cells in the hospital environment or health care settings. C. difficile adheres and colonizes in the intestinal tract with multiple adhesion factors, such as flagella, S-layer protein (SlpA), and cell wall protein (Cwp66) (4). After usage of the antibiotics, the patient's intestinal flora was disrupted by antibiotics. Thus, toxigenic C. difficile strains, which produce Toxin A and Toxin B toxins, gain the niche to self-reproduce and produce TcdA and TcdB, which cause cytoskeletal alterations that result in breaking of the tight junctions of the epithelial connection. Cytotoxic toxins translocated into the cells can cause inflammation and the accumulation of neutrophils by inducing the release of various immunomodulatory mediators from epithelial cells, which eventually leads to diarrhea and pseudomembranous colitis (5).

Cell adhesion to the intestinal cells is an essential step of the *C. difficile* infection (CDI). The cell adhesion process of C. difficile is related to S-layer proteins, which consists of S-layer protein A (SlpA) heterodimers and more than 30 cell wall proteins (CWPs). CWPs are a large family of gene products, significant homology to surface layer proteins (SLPs), such as the *slpA* gene (6). The cell wall protein 66 gene (*cwp66*, CD630_27890, molecular weight = 66 kDa) encodes the cell wall protein 66 (Cwp66) (7). The Cwp66 protein, flagellin C (FliC), flagellin D (FliD), and cell wall protein 84 (Cwp84), are major serum antigens of C. difficile (8), and play a vital role in evoking a strong immune response (9).

The Cwp66 protein contains three domains: a signal peptide (SP), three cell wall binding 2 domains (CWB2), and a variable domain. The surface-exposed domain are homologies to the autolysin CwlB of Bacillus subtilis. The Cwp66 protein has long been proposed as one of the major adhesion factors of C. difficile. Antibody raised against Cwp66 partially inhibited adherence of C. difficile to cultured cells, which suggested that Cwp66 is an adhesin (10). However, only heat-shocked bacteria exhibited binding ability, leaving adhesion activity at physiology temperature still undetermined (11). Perplexingly, RNA interference approaches have been applied to genetic interference of the *cwp66* gene. However, the results showed no statistically significant differences in the Cwp66 protein expression nor the adherence of recombinant C. difficile strains (12). Thus, further work should be carried out to elucidate the function of the *cwp66* gene.

Previously, we constructed a gene engineering toolkit based on the CRISPR-Cpf1 system (13). We applied the CRISPR-Cpf1 toolkit in the present study to construct a Δ*cwp66* mutant. Then, we identified the phenotypic changes of the Δ*cwp66* mutant and further explored the underlying mechanisms of these changes by using RNA-sequencing methods.

## RESULTS

### Verification of *cwp66* mutant.

**FIG 1:**
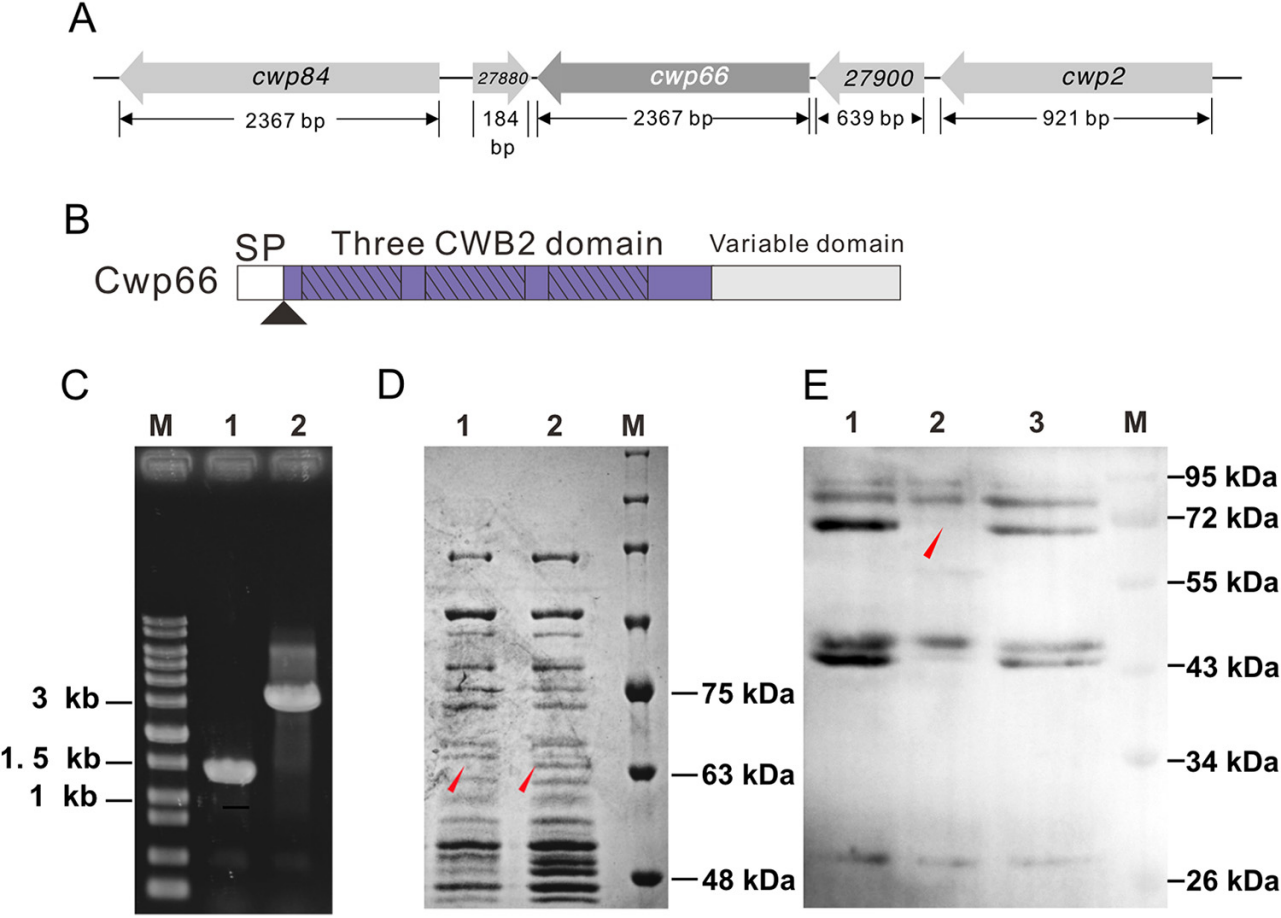
Verification of Δ*cwp66* gene mutant. (A) Gene context of the *cwp66* gene, the *cwp66* gene is flanked by CD630_27880 and CD630_27900 genes. (B) The primary structure of *cwp66* gene, it consists of a signal peptide (SP), three cell wall binding 2 domains (CWB2), and a variable domain. (C) Verification of Δ*cwp66* mutant by using diagnostic PCR. Lane 1, PCR amplicon using genomic DNA of Δ*cwp66* mutant as the template; Lane 2, PCR amplicon using genomic DNA of WT strain as the template; lane M, DNA marker (from the top to the bottom, 10 k, 8 k, 6 k, 5 k, 4 k, 3 k, 2 k, 1.5 k, 1 k, 0.75 k, 0.5 k and 0.2 k). The reduction of PCR products indicates that the coding sequence of the *cwp66* gene was deleted from the C. difficile 630 genome. (D) Verification of Δ*cwp66* mutant by using SDS-PAGE. Lane 1, cell lysate of the Δ*cwp66* mutant; Lane 2, cell lysate of the WT strain. The results showed that the Δ*cwp66* mutant strain absented a 66 kDa protein band than the WT strain (red arrows); lane M molecular weight marker (from the top to the bottom, 245 kDa, 180 kDa, 135 kDa, 100 kDa, 75 kDa, 63 kDa and 48 kDa). (E) Verification of Δ*cwp66* mutant Western blot analysis using the Cwp66 protein-specific antibodies. Lane 1, cell lysate of the WT strain; Lane 2, cell lysate of the Δ*cwp66* mutant; Lane 3, cell lysate of the ΔPaLoc strain, in which the pathogenicity locus (PaLoc) of C. difficile 630 strain was deleted, and the *cwp66* gene in the ΔPaLoc strain was intact; Lane M, molecular weight marker (from the top to the bottom, 95 kDa, 72 kDa, 55 kDa, 43 kDa, 34 kDa and 26 kDa). The red arrowhead indicates the absent the Cwp66 band.

The *cwp66* (CD630_27890) gene is located in the CWPs gene cluster, flanked by CD630_27880 and CD630_27900 genes (Fig. 1A). The *cwp66* gene consists of a signal peptide, three CWB2 homologous domains, and a variable domain (Fig. 1B) (14). The plasmid pWH55, which contains the *cwp66* gene targeting crRNA (5′-GCAGTGGGTGTATTAGCAGCTAA-3′), was conjugated into C. difficile 630 strain. The conjugation efficiency was 2.11 × 10^2^ CFU/mL-donor. The gene-editing efficiency was 100% (13). The *cwp66* gene (1,826 bp) was deleted from the genome of C. difficile 630 strain (from ATG to TAA) (Fig. 1C). The *cwp66* gene completion mutant was constructed by conjugating plasmid pZQS1 to Δ*cwp66* mutant, which contained an iLacP::*cwp66* expression cassette and denoted : :*cwp66* mutant hereafter.

As shown in Fig. 1C, the wild-type (WT) C. difficile 630 produces 3,200 bp PCR amplicon, whereas Δ*cwp66* mutant produces 1,300 bp PCR product. Further gene sequencing results confirmed that the *cwp66* gene was deleted as expected (Fig. S2A). To verify whether the Cwp66 protein was expressed in the Δ*cwp66* mutant, cell lysates of both WT and Δ*cwp66* mutant were analyzed by SDS-PAGE. A band with a molecular weight of nearly 66 kDa was missed in the Δ*cwp66* mutant compared with the WT strain (Fig. 1D), which suggested the Δ*cwp66* mutant was successfully constructed. Furthermore, antibodies against Cwp66 protein were obtained by injecting synthesized peptide (N-SGNKPKVNDTEKETK-C) into rabbits, and obtained antibodies were used in the Western blot analysis. The result showed that the Cwp66 protein was undetectable in the Δ*cwp66* mutant (Fig. 1E).

### Phenotypic analyses of the Δ*cwp66* mutant.

**FIG 2:**
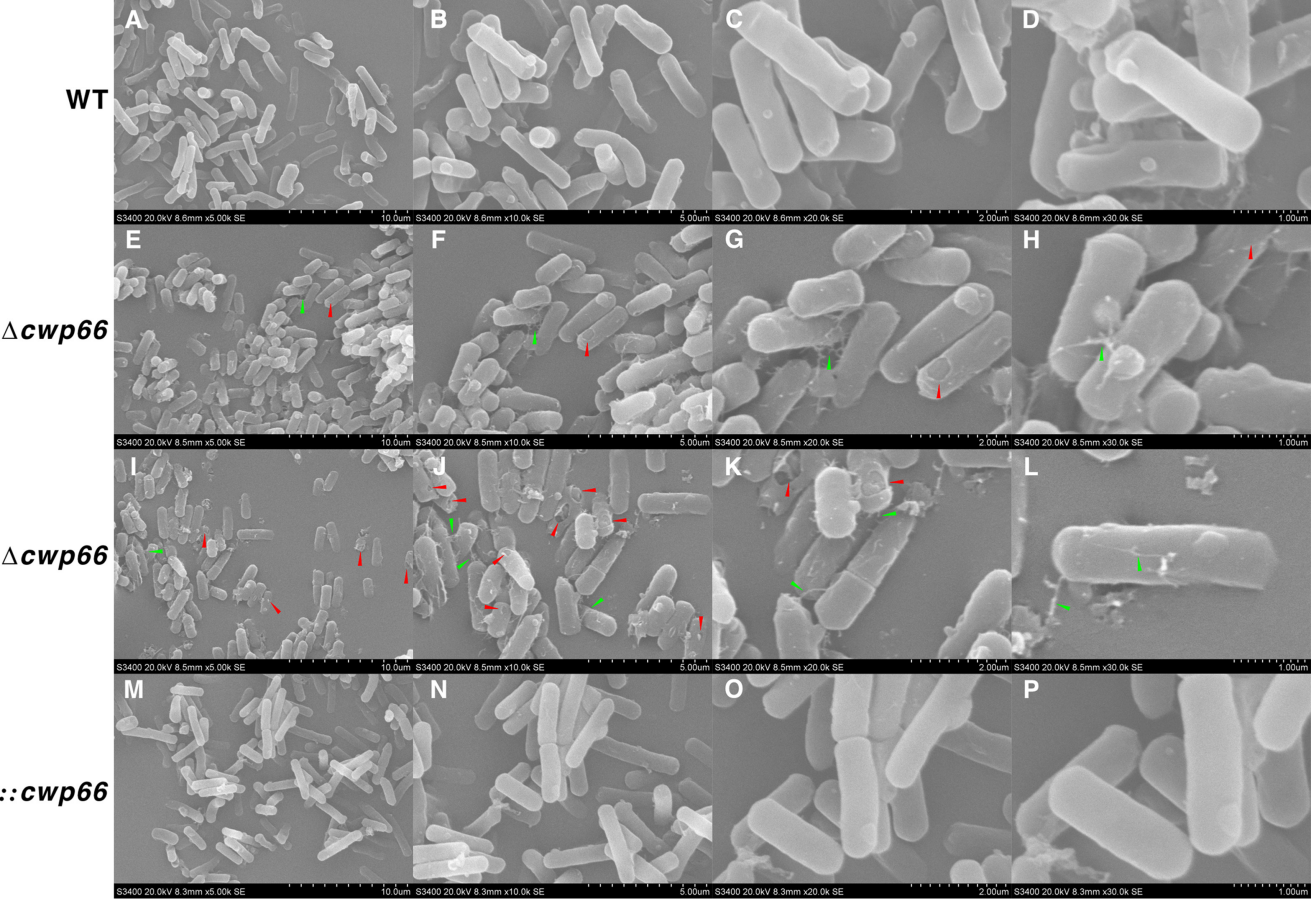
Changes in cells surface morphology of *C. difficile* strains. Cells surface morphology of the WT strain at ×5, 000 (A), ×10, 000 (B), ×20, 000 (C), and ×30, 000 (D) magnification. The first view of cells surface morphology of the Δ*cwp66* strain at ×5, 000 (E), ×10, 000 (F), ×20, 000 (G), and ×30, 000 (H) magnification (red arrows indicate cell disruption on the cell surface, green arrows indicate filamentous structure). The second view of cells surfaces morphology of the Δ*cwp66* strain at ×5, 000 (I), ×10, 000 (J), ×20, 000 (K), and ×30, 000 (L) magnification (red arrows indicate cell disruption on the cell surface, green arrows indicate filamentous structure). Cells surface morphology of the ::*cwp66* strain at ×5, 000 (M), ×10, 000 (N), ×20, 000 (O) and ×30, 000 (P) magnification.

We analyzed the phenotypes of the Δ*cwp66* mutant, including growth profile, cell adhesion ability, autolysis rate, pH sensitivity, oxygen tolerance, and antibiotic resistance. The cell surface morphologies of the WT, Δ*cwp66*, and ::*cwp66* mutant are shown in Fig. 2. The C. difficile 630 strain showed a smooth and intact cell surface (Fig. 2A to D), whereas many Δ*cwp66* mutants showed disrupted cell surface (red arrows) and production of filamentous structure (green arrows) (Fig. 2E to L). As expected, the ::*cwp66* completion mutant restored the smooth and intact cell surface like the WT strain (Fig. 2M to P). These results strongly indicated that the Cwp66 protein is vital in forming and maintaining cell surface structure. Furthermore, the concentrations of the toxins in the culture supernatant were measured, and results showed that the Δ*cwp66* mutant released more toxins into the supernatant than the WT strain (Fig. S2B). These results indicated that deletion of the *cwp66* gene altered the cell surface structure of C. difficile.

**FIG 3:**
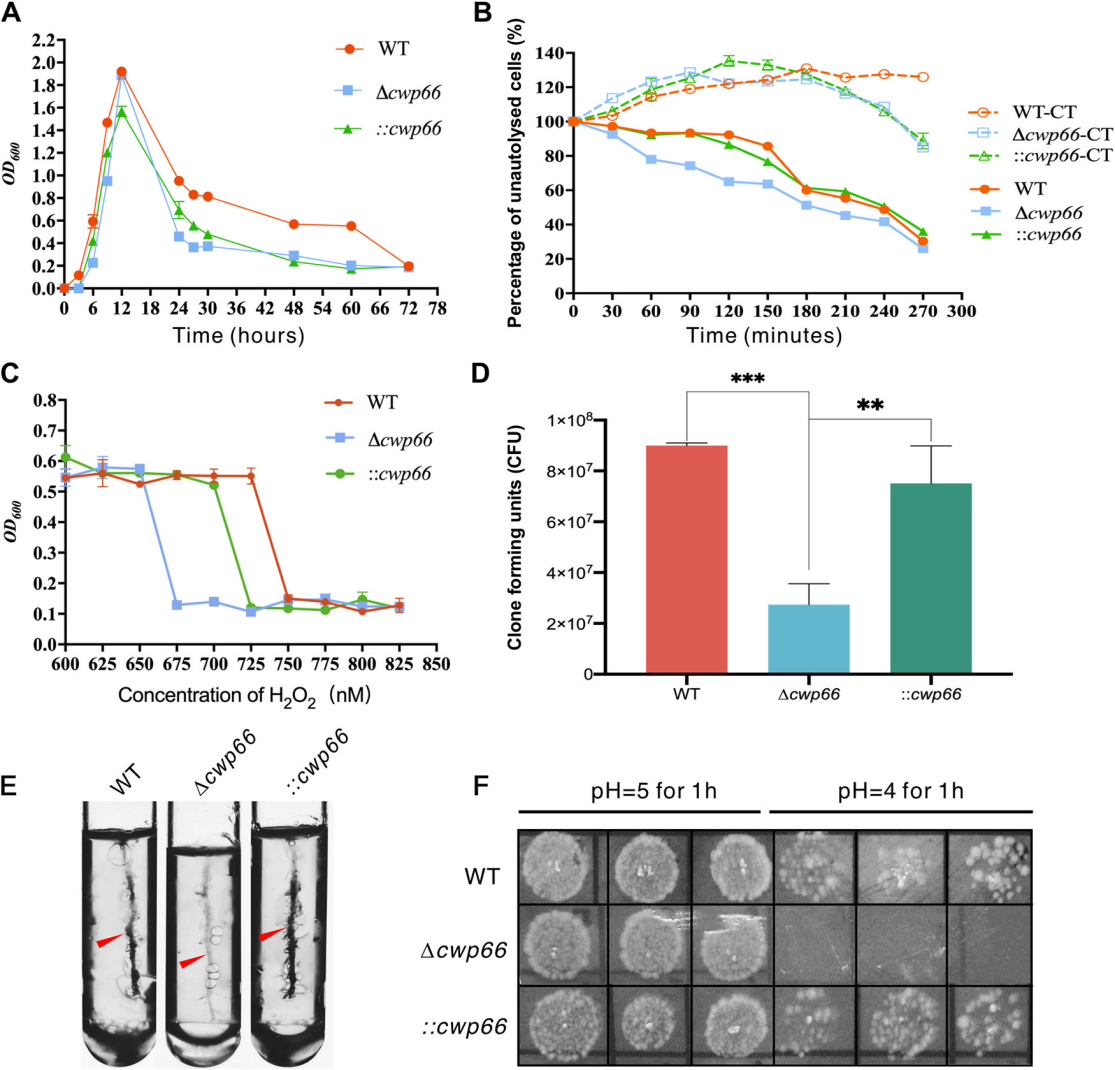
Phenotypic analysis of the Δ*cwp66* mutant. (A) Growth curves of WT, Δ*cwp66* and ::*cwp66* mutants, the horizontal coordinate is incubation time (hours), the vertical coordinate is cell turbidity (OD_600_). (B) Cell autolysis rate of WT, Δ*cwp66* and ::*cwp66* mutants after treated with Triton X-100, the horizontal coordinate is treatment duration of Triton X-100, the vertical coordinate is the percentage of unautolysed cells, the “-CT” suffix means untreated control. (C) Changes of cell tolerance to peroxide, the horizontal coordinate is the concentration of H_2_O_2_, the vertical coordinate is the cell turbidity (OD_600_). (D) Changes in cell adhesion ability of WT, Δ*cwp66*, and ::*cwp66* mutants under anaerobic condition, the horizontal coordinate indicates different strains, the vertical coordinate indicates the CFU counts of cells adhered to Caco-2 cells. The adhesion ability of Δ*cwp66* was decreased significantly (decreased more than 3-fold compared with the WT strain), and the ::*cwp66* mutant restored 83% of adhesion ability compared with the WT strain. (E) Comparison of motility of the WT, Δ*cwp66*, and ::*cwp66* mutants, the motility of the Δ*cwp66* mutant strain was slightly decreased than that of the WT and the ::*cwp66* stain restored cell motility. (F) Growth of WT, Δ*cwp66*, and ::*cwp66* mutants on BHI plate at pH = 4 and 5. The Δ*cwp66* strain failed to grow in BHI solid medium at pH = 4. In comparison, the WT and ::*cwp66* strains grew well on the BHI medium at pH = 4. Student’s *t* test was used to compare the differences between groups, and the results were expressed as mean ± standard deviation, with a test level of α = 0.05. **, *P* < 0.01; ***, *P* < 0.001.

The cell surface structure is often associated with physiological characteristics, such as growth profile, cell autolysis, pH, and oxygen tolerance. We first analyzed the growth profiles of the Δ*cwp66, ::cwp66* mutants and the WT strain. As shown in Fig. 3A, the Δ*cwp66* mutant showed a slightly slower growth initiation rate (0 h ∼ 12 h) and a much faster cell lysis rate (12 h ∼ 72 h), whereas the ::*cwp66* mutant partially restored the cell growth profile of the WT strain. We next measured changes in the autolysis rate of the three strains, the WT, Δ*cwp66*, and ::*cwp66* strains reached 50% of cells lysis at 240 min, 180 min, and 240 min, respectively (Fig. 3B), which indicated that the cell lysis rate of the Δ*cwp66* mutant was higher. We also measured the tolerance of Δ*cwp66* to peroxide (H_2_O_2_), the Δ*cwp66* strain was more sensitive to H_2_O_2_ than the WT strain (675 nM versus 750 nM), and the ::*cwp66* strain partially restored the H_2_O_2_ resistant profile (725 nM) (Fig. 3C).

The *cwp66* gene has been predicted to be a cell adhesion factor (10, 12). Thus, we measured the cell adhesion ability of the Δ*cwp66* mutant strain compared with the WT and the ::*cwp66* strains (15). In the anaerobic condition (90% N_2_ and 10% H_2_), the adhesion ability of Δ*cwp66* decreased more than 3-fold (compared with the WT strain) (Fig. 3D). As expected, the ::*cwp66* mutant restored 83% of adhesion capability compared with the WT strain (Fig. 3D). Then the cell motility of the WT, Δ*cwp66*, and ::*cwp66* strains was measured. The result showed that the motility of the Δ*cwp66* mutant strain was slightly decreased than that of the WT and the ::*cwp66* strain (Fig. 3E, red arrows). Finally, the pH sensitivity of the strains was measured. At pH = 4, the Δ*cwp66* strain failed to grow in BHI solid medium. In comparison, the WT and ::*cwp66* strains grew well at the same pH setting, which means pH tolerance of the Δ*cwp66* strain was decreased and the ::*cwp66* strain restored pH tolerance (Fig. 3F).

### Antibiotic resistance profiles of Δ*cwp66* mutant.

Phenotypic studies of Δ*cwp66* mutant strains revealed altered extracellular structure, cell adhesion ability, and pH tolerance in Δ*cwp66* mutant strains. The S-layer protein determines antibiotic resistance in bacteria Staphylococcus aureus (16). However, whether Cwp66 is related to the antibiotic resistance of C. difficile is still unclear. Next, we investigated the resistance profiles of the WT, Δ*cwp66*, the ::*cwp66* strains to the commonly used clinical antibiotics (e.g., metronidazole and vancomycin).

**FIG 4:**
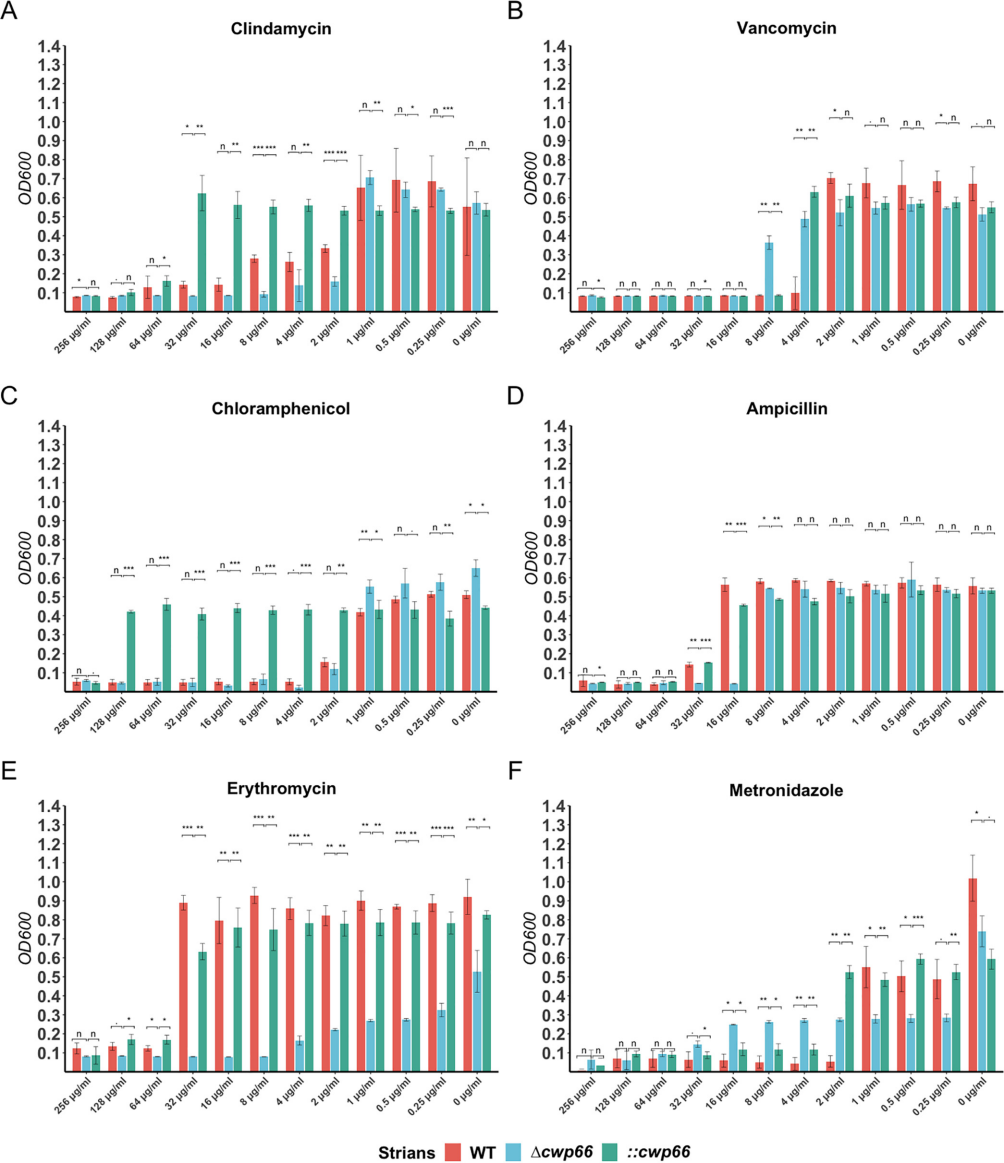
Changes of resistance profiles to antibiotics of the Δ*cwp66* mutant. The vertical coordinate is the value of OD_600_ and the horizontal coordinate is the antibiotic concentration (μg/mL). The red, light blue, and light green bars indicate the OD_600_ values of WT, Δ*cwp66*, and ::*cwp66* strain at different antibiotic concentrations. The Δ*cwp66* mutant is more sensitive than the WT strain to clindamycin, ampicillin, and erythromycin but more resistant than the latter to vancomycin and metronidazole. Except for chloramphenicol (due to plasmid born chloramphenicol transacetylase gene), overexpression of the *cwp66* gene in the Δ*cwp66* mutant restored antibiotics resistant profiles for all tested antibiotics. Student’s *t* test was used to compare the differences between groups, and the results were expressed as mean ± standard deviation (*n* = 3), with a test level of α = 0.05. n, *P* > 0.1; ., *P* > 0.05; *, *P* < 0.05; **, *P* < 0.01; ***, *P* < 0.001.

The Δ*cwp66* mutant was more sensitive than the WT strain to clindamycin (Fig. 4A), ampicillin (Fig. 4D), and erythromycin (Fig. 4E), but more resistant than the latter to vancomycin (Fig. 4B) and metronidazole (Fig. 4F). Except for chloramphenicol, overexpression of the *cwp66* gene in the Δ*cwp66* mutant restored antibiotic resistant profiles for all tested antibiotics (Fig. 4). The ::*cwp66* mutant exhibited high resistance (MIC = 256 μg/mL) to chloramphenicol due to the completion plasmid contained a chloramphenicol acetyltransferase (CAT) gene. Compared with the WT strain, the Δ*cwp66* mutant strain showed no change in tolerance to norfloxacin, d-cycloserine, thiamphenicol, chloramphenicol (Fig. 4C), tetracycline, amoxicillin, and streptomycin (Fig. S1).

### Comparative transcriptomic analyses of gene expression profiles of the WT and the Δ*cwp66* mutant.

We further compare the expression profiles of the Δ*cwp66* mutant and the WT strain at the exponential growth phase. The sequencing library was sequenced on a NextSeq 500 platform (Illumina) by Shanghai Personal Biotechnology Co. Ltd. In total, nine genes were upregulated, 12 were downregulated (Table 1), and the expression intensity of 3,283 genes was not changed. As expected, compared with the WT strain, the *cwp66* gene was nearly undetectable. Interestingly, no transcripts of *gpr* (GPR endopeptidase) were detected either. The RNA-sequencing data highlighted that the *cwpV* gene (CD630_05140) upregulated 22.90-fold in Δ*cwp66* mutant and CD630_02170 (nitroreductase family protein), FliA/WhiG family RNA polymerase sigma factor (CD630_02660) genes downregulated 10.11- and 7.35-folds, respectively.

**TABLE 1:** Differentially expressed genes of the Δ*cwp66* mutant compared with the WT strain

Gene_ID	Name	WT	Δ*cwp66*	Fold change	Regulation
CD630_02880	PTS system, mannose/fructose/sorbose IIC component	137.6666411	811.0215327	5.891198667	Up regulation
CD630_02890	PTS system, mannose/fructose/sorbose IID component	168.1440674	1166.45511	6.937236192	Up regulation
CD630_03050	Amidohydrolase	134.3641853	518.7391778	3.860695294	Up regulation
CD630_05140	*cwpV* (cell wall-binding protein *CwpV*)	1215.613506	27836.75169	22.89934386	Up regulation
CD630_16730	Conjugal transfer protein TraX	23.22251014	69.27760194	2.98320903	Up regulation
CD630_20990	Molybdopterin-dependent oxidoreductase	692.7271125	1569.057103	2.265043586	Up regulation
CD630_23310	*mtlD* (mannitol-1-phosphate 5-dehydrogenase)	255.5612433	748.7770499	2.929931942	Up regulation
CD630_27900	PIG-L family deacetylase	596.7339989	1318.281645	2.20916128	Up regulation
CD630_34900	*spoIIE* (stage II sporulation protein E)	6.547804237	36.47643253	5.570788498	Up regulation
CD630_01670	Sigma 54-interacting transcriptional regulator	47.0362042	11.5697502	4.065446815	Down regulation
CD630_02170	Nitroreductase family protein	13.33166109	1.318271326	10.112987249	Down regulation
CD630_02660	FliA/WhiG family RNA polymerase sigma factor	23.64113308	3.217007852	7.348795591	Down regulation
CD630_07610	DEAD/DEAH box helicase	1850.480638	820.3464767	2.255730584	Down regulation
CD630_14080	*ddl* (d-Alanine-d-Alanine ligase)	232.7895116	61.51466946	3.784292655	Down regulation
CD630_16970	*ribH* (6.7-dimethyl-8-ribityllumazine synthase)	511.165506	185.3023467	2.758548473	Down regulation
CD630_21180	*thrC* (threonine synthase)	1657.586309	662.9149163	2.500451063	Down regulation
CD630_22770	Class II aldolase/adducin family protein	48.5436091	23.35252607	2.078730539	Down regulation
CD630_24700	*gpr* (GPR endopeptidase)	12.77641842	0	NA^*a*^	Down regulation
CD630_24790	tRNA threonylcarbamoyladenosine dehydratase	97.63792658	22.97017697	4.250638852	Down regulation
CD630_27890	*cwp66* (cell wall-binding protein Cwp66)	3625.652758	2.6257237	1380.820676961	Down regulation
CD630_28070	*ruvC* (crossover junction endodeoxyribonuclease RuvC)	18.93350303	2.769434328	6.836595769	Down regulation

a NA, not applicable.

Four genes, CD630_02890 (PTS system mannose/fructose/sorbose family transporter subunit IID), CD630_02880 (PTS system, mannose/fructose/sorbose IIC component), CD630_34900 (*SpoII*E), and CD630_03050 (amidohydrolase), were upregulated 6.94-, 5.89-, 5.57- and 3.86-fold, respectively. The CD630_28070 (RuvC crossover junction endodeoxyribonuclease), CD630_24790 (tRNA threonylcarbamoyladenosine dehydratase), CD630_01670 (Sigma 54-interacting transcriptional regulator), and CD630_14080 (*ddl*, d-Alanine-d-Alanine ligase) genes were downregulated by 6.84-, 4.25-, 4.07-, 3.78-fold, respectively (Table 1).

### Kyoto Encyclopedia of Gene and Genomes and gene ontology analyses.

**FIG 5:**
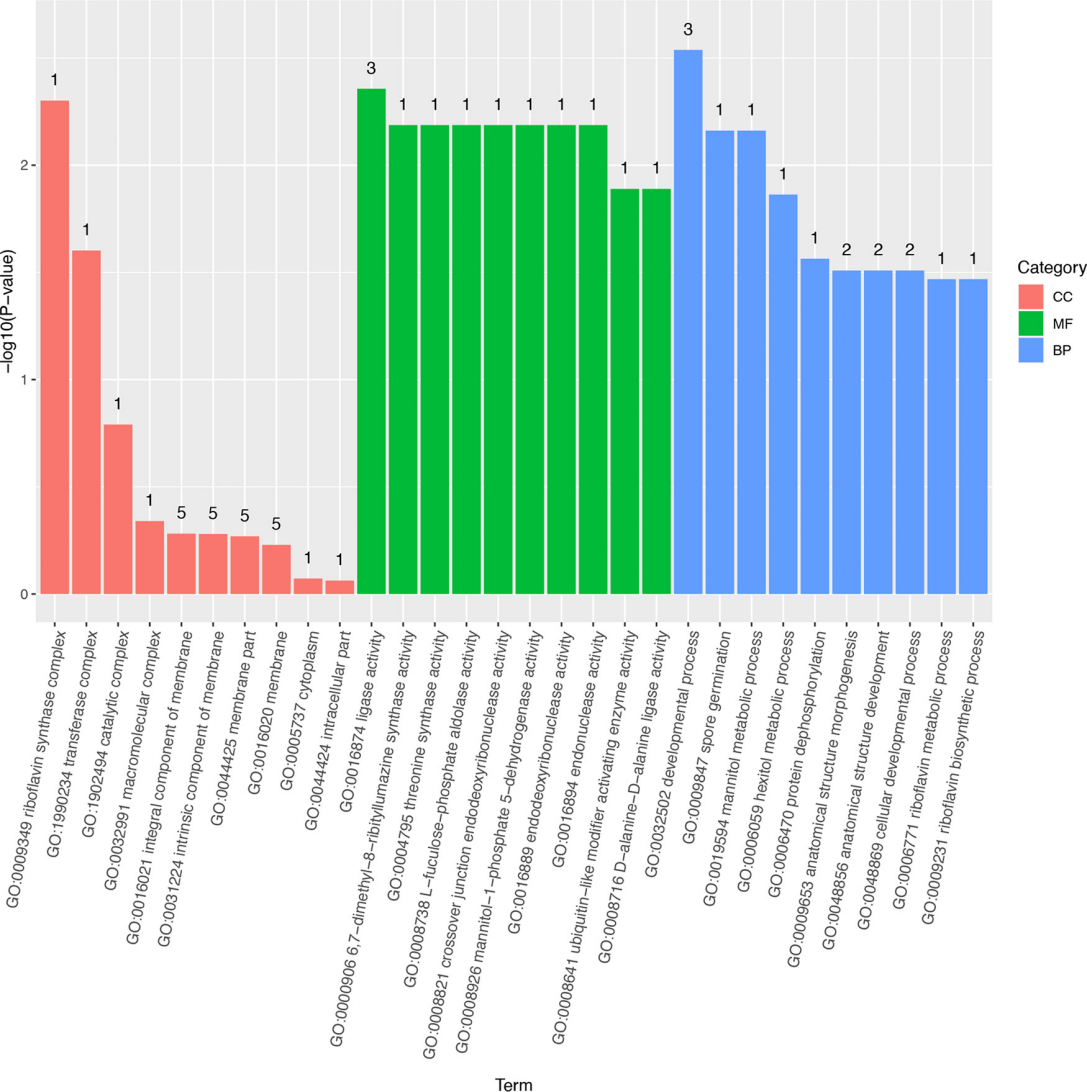
Gene ontology classification. The horizontal coordinate is the term of GO level 2, the vertical coordinate is the –log_10_(*P*-value) enriched for each term, and the number on the column is the number of differential genes enriched to the corresponding term.

Afterward, we performed gene ontology (GO) and Kyoto Encyclopedia of Gene and Genomes (KEGG) pathway enrichment analysis using topGO, based on nucleotide annotation for WT versus Δ*cwp66* differentially expressed genes (17). Accordingly, 14, 95, and 135 terms were produced for cellular component (CC), molecular function (MF), and biological process (BP) category, respectively. The most significantly enriched gene set of CC, MF, and BP were riboflavin synthase complex (GO:0009349), ligase activity (GO:0016874), and developmental process (GO:0032502), respectively (Fig. 5).

**FIG 6:**
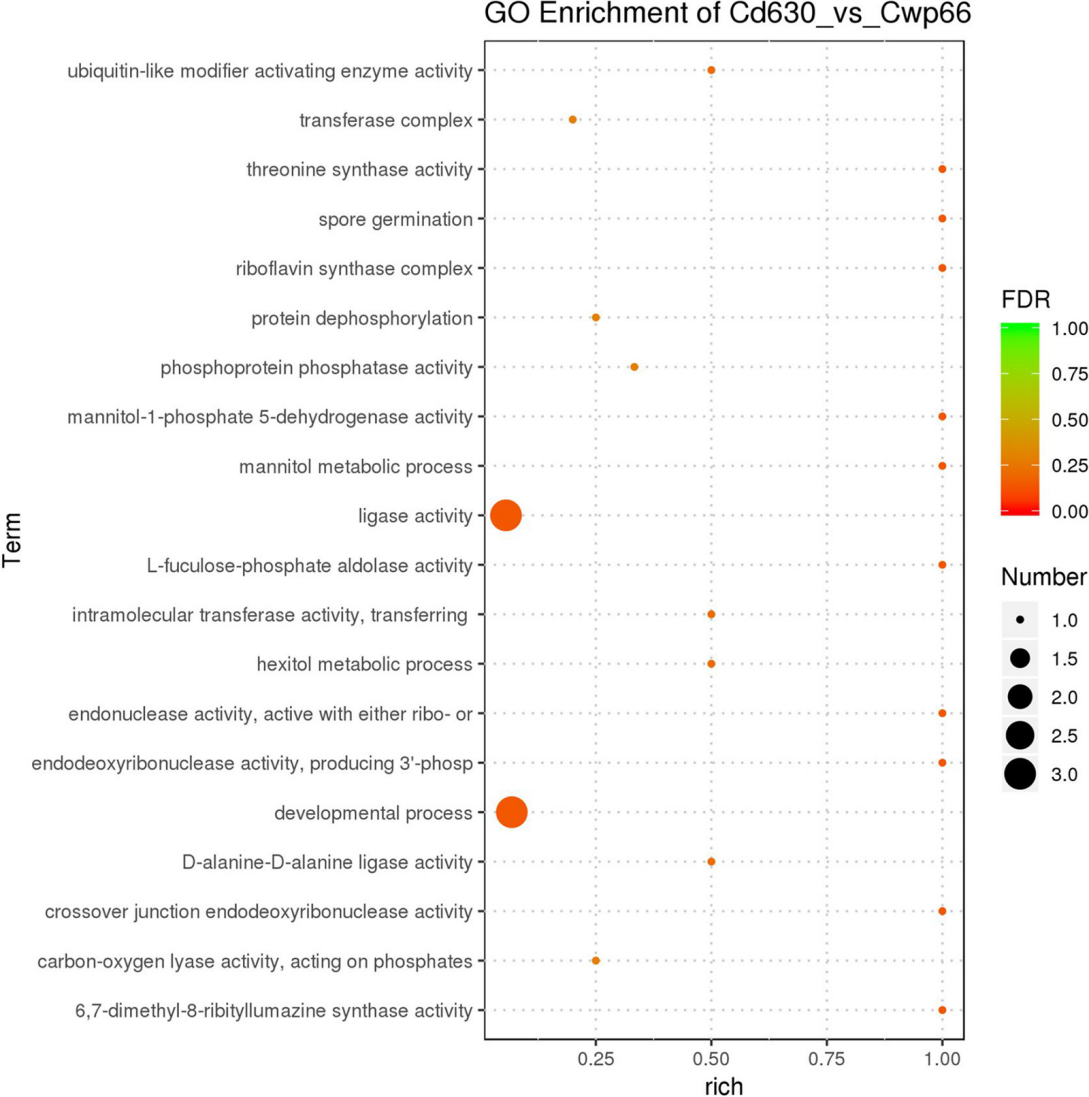
The GO enrichment analysis. The 20 most significantly enriched GO Term entries are displayed. The vertical coordinate is the GO term entries; the horizontal coordinate is the Rich factor, and the size of the bubbles indicates the number of differential genes enriched to the term, and the color indicates the false discovery rate (FDR) value of the pathway.

In the CC, riboflavin synthase complex (GO:0009349), transferase complex (GO:1990234), catalytic complex (GO:1902494), macromolecular complex (GO:0032991), an integral component of the membrane (GO:0016021), and an intrinsic component of the membrane (GO:0031224) were the dominant subcategories. As MF category was concerned, ligase activity (GO:0016874), 6,7-dimethyl-8-ribityllumzaine synthase activity (GO: 000906), threonine synthase activity (GO: 0004795), L-fuculose-phosphate aldolase activity (GO: 0008738), crossover junction endodeoxyribonuclease activity (GO:0008821), mannitol-1-phosphate-5-dehydrogenase activity (GO:0008926), endodeoxyribonuclease activity (GO: 0016889), endonuclease activity (GO:0016894), ubiquitin-like modifier activation enzyme activity (GO:0008641), and d-Alanine-d-Alanine ligase activity (GO: 0008716) were the top 10 subcategories. When BP was considered, developmental process (GO: 0032502), spore germination (GO:0009847), mannitol metabolic process (GO: 0019594), and hexitol metabolic process (GO: 006059) were the top four subcategories (Fig. 5). The bubble map showed that the ligase activity and development process were the most affected gene cluster in the Δ*cwp66* mutant compared with the WT strain (Fig. 6).

**FIG 7:**
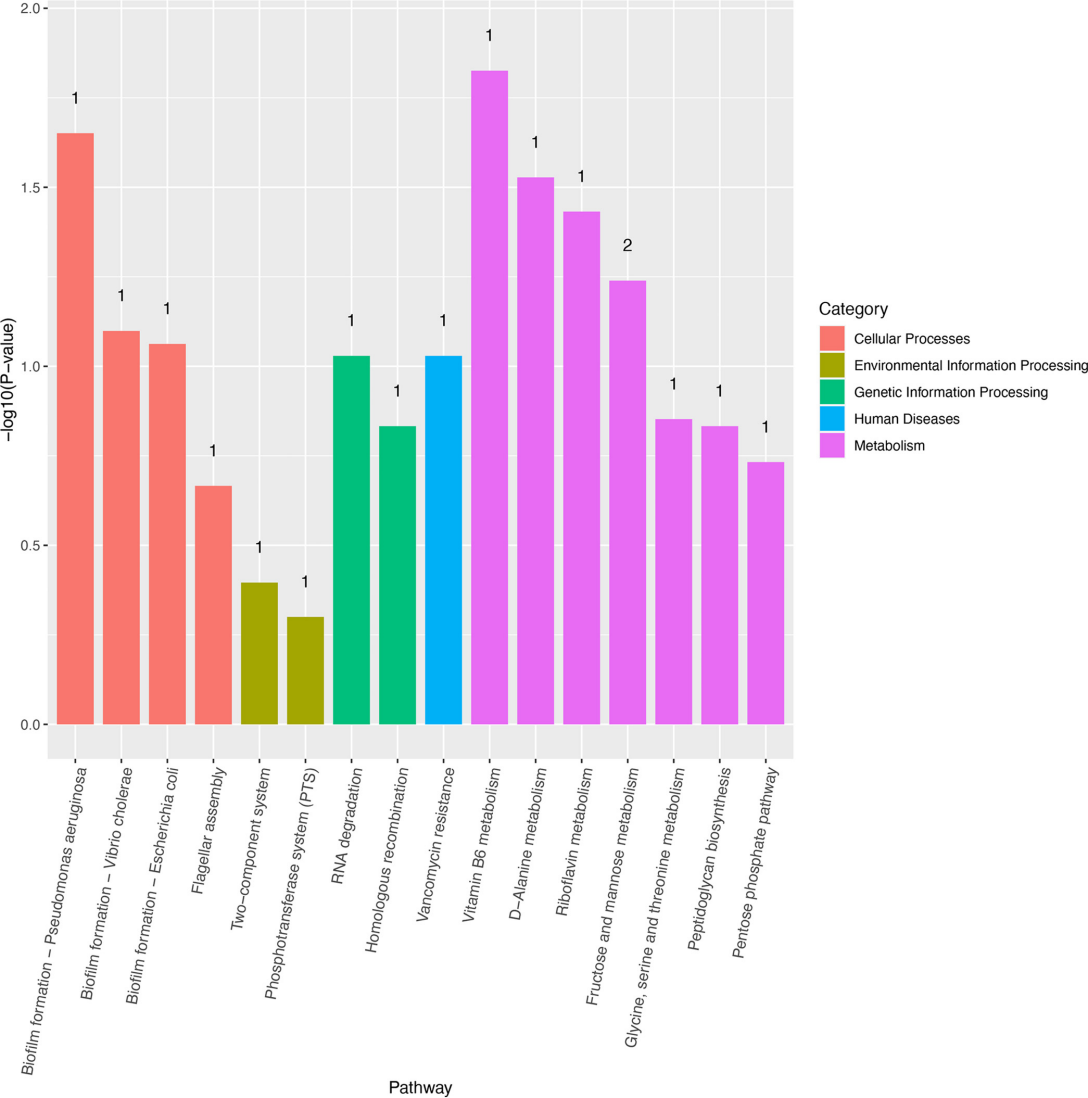
Annotation result against the KEGG database. The horizontal coordinate is the pathway's name; the vertical coordinate is the –log_10_ (*P*-value) enrichment for each pathway, and the number on the column is the number of differential genes enriched to the corresponding term.

**FIG 8:**
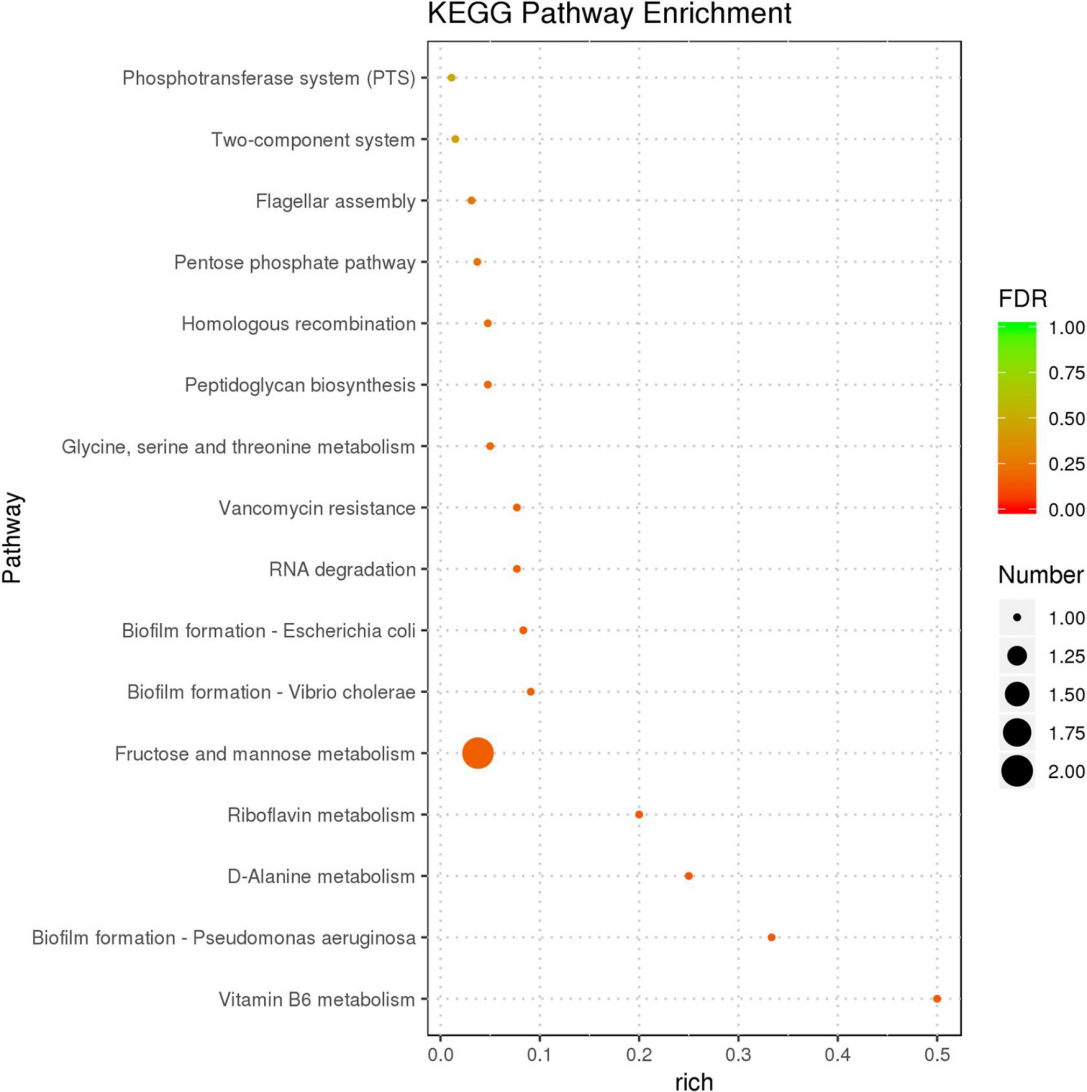
The GO enrichment analysis. The 16 most significantly enriched KEGG pathways are displayed. The vertical coordinate is the KEGG pathway; the horizontal coordinate is the rich factor, and the size of the bubbles indicates the number of differential genes enriched to the pathway, and the color indicates the FDR value of the pathway.

The KEGG is a biological pathway analysis database. The KEGG analysis showed that differentiated expressed genes were annotated into 16 known KEGG pathways. Metabolism pathways were the largest subcategories involving seven unigenes including Vitamin B6, d-Alanine, Riboflavin, Fructose and mannose metabolism, glycine, serine and threonine metabolism, peptidoglycan biosynthesis, and pentose phosphate pathway, followed by cellular processes consisting of four unigenes including biofilm formation and flagellar assembly. Moreover, two unigenes were involved in genetic information processing (RNA degradation and homologous recombination), and two unigenes were involved in environmental information processing (two-component and phosphotransferase systems). More importantly, the vancomycin resistance pathway was enriched in the Δ*cwp66* mutant compared with the WT strain, consistent with the observation of the increase of vancomycin resistance of the Δ*cwp66* mutant (Fig. 4 and 7). Furthermore, the bubble map showed that the deletion of the *cwp66* gene also exerts dominant effects on fructose and mannose metabolism (Fig. 8). The sole carbon source experiment confirmed that the mannose and fructose utilization capabilities in the Δ*cwp66* mutant were slightly decreased compared with the WT strain. Interestingly, mannitol utilization in the Δ*cwp66* mutant was impaired (Fig. S3).

## DISCUSSION

C. difficile is a Gram-positive, spore-forming anaerobic bacteria that is one of the leading causes of antibiotic-associated diarrhea in developed countries (18). Ingestion of the C. difficile spores can lead to the asymptomatic carriage of clinical symptoms ranging from mild or severe diarrhea to life-threatening pseudomembranous colitis (PMC) (19). In the present study, we constructed a Δ*cwp66* mutant using the CRISPR-Cpf1 gene-editing tool (13). The gene sequencing, SDS-PAGE, and Western blot results validated that the *cwp66* gene and its encoding Cwp66 protein were absent in the Δ*cwp66* mutant. As expected, the adherence ability of the Δ*cwp66* mutant was decreased significantly under anaerobic conditions. The stress-tolerance abilities of Δ*cwp66* mutant to Triton X-100 (cell lysis rate), acidic environment (pH = 4), and oxidative stress (H_2_O_2_) were decreased. Moreover, the antibiotic resistance profile was significantly changed in the Δ*cwp66* mutant.

The initiation step of CDI was adhesion of C. difficile strain to the intestine cells. Studies of proteins located on the surface of C. difficile strain have underlined the multi-factorial involved in C. difficile adhesion to the intestine cells. The SlpA, Cwp66, Cwp2, and CD_0873 proteins were highlighted as adhesion factors. Although the Cwp66 protein plays a vital role in C. difficile pathogenicity, with our limited knowledge, no *cwp66* complete gene mutant has been yet constructed and characterized (19). In the present work, as we constructed a Δ*cwp66* mutant, the phenotypic and transcriptome changes of the Δ*cwp66* mutant compared with the WT strain were studied. Cwp66 is suggested as a cell adhesion using immunological methods, and antibodies raised against Cwp66 inhibited adherence of C. difficile to cultured cells (10). The present gene deletion and completion experiments added direct evidence that the *cwp66* gene plays an essential role in cell adhesion, consistent with previous studies (20).

The cell wall proteins embedded in the bacteria cell wall comprise a polysaccharide polymer cross-linked by peptides to reinforce its physical strength (21). It provides a barrier to protect the bacterial cell against external forces causing cell lysis (e.g., mechanical and osmotic forces) (5). The Cwp66 protein is a vital component of the S-layer. The S-layer has been proposed to protect environmental stress or virulence factors from the host immune system (22). Therefore, it is likely that the deletion of the *cwp66* gene influenced C. difficile cell wall’s physical strength by altering the S-layer composition (disruption on the cell surface of Δ*cwp66* mutant, Fig. 2E to L), thus leading to increased cell lysis rate, more susceptible to peroxide, and changes in tolerance to acidic environments.

To further reveal the underlying mechanism of phenotypic changes in Δ*cwp66* mutant, we used paired-end RNA-seq technology to study the transcriptomic difference between two strains. The results showed that nine genes were upregulated, and 12 were downregulated in the Δ*cwp66* mutant, which was in line with the phenotypic changes of the Δ*cwp66* mutant. We first noticed that the gene expression intensity of another adhesion CwpV was increased sharply (up to 23 folds). The CwpV is a dominant member of the CWP family, it contains (i) a putative N-terminal cell wall binding domain (CWB2); (ii) an unknown function domain; and (iii) nine repeats of 120 amino acids each (23). The CwpV protein has been proposed as a putative adhesin based on homology to a known hemagglutinin from Salmonella *typhimurium* (24). Moreover, the CwpV accounts for almost 15% of S-layer associated protein in 5% of C. difficile cells. It promotes auto-aggregation of cells in both liquid and solid media (23), similar to those reported in mouse models of colonization (25). Together, these findings suggest that the CwpV may play a role in the host colonization, and we reasoned that the reduction in cell adhesion ability due to deletion of the *cwp66* gene might partially be compensated by the overexpression of the *cwpV* gene. Furthermore, the two genes may be co-regulated by an unknown mechanism which needs further study.

Furthermore, in the Δ*cwp66* mutant, the mannose/fructose/sorbose transporter IIC (CD630_02880) and IID (CD630_02890) subunit of the phosphotransferase system (PTS) were upregulated 5.89- and 6.94-fold, indicating that the phosphotransferase system was affected. The PTS has catalytic and regulatory activities, catalyzing the uptake of multiple carbon sources, phosphorylation, and toxin uptake (26). The mannose/fructose/sorbose IIC and IID subunits catalyze the transfer of phosphoenolpyruvate (PEP) phosphate groups to the carbon source and participate in transferring mannose/fructose/sorbose to the C. difficile 630 cells. Interestingly, the sole carbon source experiment showed that the mannose and fructose utilization capabilities in Δ*cwp66* decreased slightly, whereas mannitol utilization in the Δ*cwp66* mutant was impaired (Fig. S3). The transcriptome results showed that the expression levels of mannitol utilization-related genes such as mannitol dehydrogenase and fructokinase were not changed (27). Thus, we reasoned that Δ*cwp66* knockout affects the mannitol transport machinery (GO:0015797) or upstream regulator through an unknown mechanism (28), which needs further work to elucidate.

Vancomycin is a glycopeptide antibiotic that inhibits bacterial cell wall synthesis at an earlier stage than the beta-lactam antibiotic. It acts by binding to pentapeptide d-Alanine-d-Alanine residues, blocking the cross-bridge linkage between pentapeptide and pentaglycine, impeding bacterial cell wall synthesis, and acting as a bacteriostatic and bactericidal agent (29). The KEGG enrichment analysis revealed that the d-Alanine metabolism pathway strain was highly enriched in the Δ*cwp66* mutant, and the d-Alanined-Alanine-ligase expression was downregulated 3.78-fold. These results suggested that the *cwp66* gene was associated with vancomycin resistance via d-Alanine-d-Alanine-ligase. In Staphylococcus aureus, vancomycin resistance is a multi-gene participating process involving *vraTSR*, *graSR*, *walKR*, *stk1/stp1*, *rpoB*, *clpP*, and *cmk* genes. Our study revealed that the *cwp66* gene was involved in vancomycin resistance of C. difficile through a potential mechanism related to influence the expression of d-Alanine-d-Alanine ligase and/or effects on the VanS/VanR two-component system, which enriched in KEGG analysis (Fig. 7 and 8).

In summary, Cwp66 protein is a vital adhesion factor of C. difficile. The deletion of the *cwp66* gene resulted in decreased cell adhesion ability and cell motility, increased cell lysis rate, more susceptibility to peroxide; changes in tolerance to acidic environments, antibiotic resistance profiles, and impaired mannitol transport machinery. Further transcriptome analysis showed that (i) *cwp66* and *cwpV* genes were correlated in transcription level; (ii) the *cwp66* gene was involved in vancomycin resistance of C. difficile by influencing the expression of d-Alanine-d-Alanine ligase and/or effects on the VanS/VanR two components system; and (iii) the mannose/fructose/sorbose IIC and IID subunits of PTS system were affected in Δ*cwp66* mutant. Together, these results suggested that Cwp66 protein plays a vital role in cell adhesion, cell motility, stress resistance, antibiotic resistance, and mannitol transportation in C. difficile.

## MATERIALS AND METHODS

### Bacterial strains and plasmids construction.

All Escherichia coli and C. difficile strains used are listed in Table S1. NEBExpress (New England Biolabs) competent cells were used for molecular cloning and plasmids construction. Plasmids were conjugated into C. difficile, and the E. coli CA434 strain was used as the plasmid donor (13). Plasmids were transformed into E. coli competent cells by using the heat shock method and the transformants were cultured in Luria-Bertani (LB) medium with the addition of ampicillin (100 μg/mL), chloramphenicol (6 μg/mL), or kanamycin (50 μg/mL) when required. The C. difficile strain was incubated in brain heart infusion (BHI) medium (supplemented with 5 g/L yeast and 1 g/L L-cycloserine) at 37°C in an anaerobic chamber (30). BHI medium was supplemented with thiamphenicol (15 μg/mL), d-cycloserine (250 μg/mL), cefoxitin (8 μg/mL), and lactose (40 mM) when appropriate.

All plasmids and primers used in this study are listed in Table S1 (plasmids) and Table S2 (primers). DNA cloning was performed using standard PCR protocol (31), and Phanta Max Super-Fidelity DNA polymerase (P505-d1, Vazyme Biotech Co., Ltd., Nanjing, China) was used. DNA assembly was carried out by using the T5 exonuclease DNA assembly method (TEDA) (32). Gene targeting plasmid was constructed by the one-step-assembly (OSA) method. All the primers for constructing single-gene-targeting plasmids were designed by the OPF algorithm (13). The GC content of spacers was set between 39% and 52%, and the length of homology arms and the overlap region for the assembly was set to ∼500 bp and 25 bp, respectively (13).

The plasmid pWH55 was designed to delete the 1,833-bp *cwp66* gene (CD_27890). The sRNAP::crRNA-*cwp66* fragment, with the specific spacer 5′-GCAGTGGGTGTATTAGCAGCTAA-3′ (PAM sequence: 5′-TTTA-3′), was amplified with primers YW3105/YW3304. Homology arms *cwp66*-Up-arm and *cwp66*-Down-arm were amplified from the C. difficile 630 gDNA with primer pairs of YW3305/YW3306 and YW3307/YW3308, respectively. The three fragments generated above were assembled with *Btg*ZI-linearized pWH34 to generate pWH55. Primer pair YW2369/YW2370 was designed to detect the deletion of *cwp66*. The PCR amplicons of WT and *cwp66* deletion mutant were 3,180 bp and 1,350 bp, respectively (13).

To construct a completion mutant of *cwp66* gene (::*cwp66*), 1,363 bp lactose inducible promoter was amplified from pWH34 plasmid using primer pair WH544/545, and the *cwp66* gene was amplified from C. difficile 630 genome using primer pair WH546/547. The *iLacP* and *cwp66* amplicons were assembled to *Bam*H I linearized pMTL83151 plasmid to obtain *cwp66* gene overexpression plasmid pZQS1 (Table S1 and S2) by using the TEDA method (32).

The Clostridium difficile minimal medium (CDMM) was prepared according to Muhammad Ehsaan et al. (33), except 20% (wt/vol) of fructose (CAS:57-48-7), mannose (CAS:3458-28-4), glucose (CAS:50-99-7), or mannitol (CAS:69-65-8) was supplemented as the sole carbon source. The 1% (vol/vol) inoculation of C. difficile strain (WT, Δ*cwp66* or ::*cwp66*) was added to 5 mL CDMM medium (supplemented with different carbon source). Then, tubes were incubated anaerobically at 37°C. Sampling was carried out at 6-h intervals for 72 h. The OD_600_ values were measured by a cell density meter (Ultrospec 10, Amersham Biosciences, GE), and then the growth curves were plotted using Prism 6.0 software (GraphPad Software, Inc).

### Antibiotics susceptibility of Δ*cwp66* mutant.

The antibiotic susceptibility of the WT, Δ*cwp66*, and ::*cwp66* strains to antibiotics (norfloxacin, d-cycloserine, thiamphenicol, tetracycline, amoxicillin, streptomycin, clindamycin, vancomycin, chloramphenicol, ampicillin, erythromycin, and metronidazole) was determined by the serial gradient dilution method (34). C. difficile was inoculated (5% inoculation rate) into 96-well plates containing 150 μL of BHI with the proper concentration of antibiotics in each well, after incubated overnight in 96-well plates anaerobically, the absorbance values at 600 nm (OD_600_) were measured by using a spectrophotometer (Varioskan LUX, Thermo Fisher). The OD_600_ values greater than or equal to 0.1 were considered the growth of C. difficile strain. In the ::*cwp66* mutant, 40 mM lactose was added to the BHIS medium to induce the expression of the *cwp66* gene (35).

### Toxin expression of the Δ*cwp66* mutant (toxin ELISA).

WT and Δ*cwp66* mutant strains were incubated in BHI medium until the OD_600_ = 1, and the cells were removed twice by centrifugation at 3,440 × *g* for 5 min. The supernatants collected and the concentration of TcdA and TcdB were determined by enzyme-linked immunosorbent assays (ELISAs) by following the instruction of the Shanghai Fankel ELISA kit (F5181-B).

### Adhesion assay.

The human colon carcinoma cell line Caco-2 (Haixing Biosciences, TCH-C146) was cultured in Dulbecco’s Modified Eagle’s Medium-high glucose (DMEM) supplemented with 20% fetal bovine serum as recommended by the producer. Moreover, 100 IU/mL of penicillin and 100 mg/mL streptomycin were also added into the medium to inhibit the growth of bacteria. After 3 days of incubation at 37°C in a 5% carbon dioxide atmosphere, cells grown as confluent monolayers (approximately 2.58 × 10^6^ cells per well) were cultured in antibiotic and serum-free medium for about 24 h, and then were transferred into the anaerobic chamber for adherence assays. Meanwhile, 1 mL of C. difficile 630 WT, Δ*cwp66*, and ::*cwp66* strains were harvested by centrifugation at 1,530 × *g* for 3 min during the exponential phase (OD_600_ = 0.6 ∼ 0.8), washed once with 1 mL of phosphate-buffered saline (PBS), and then centrifuged again to collect the C. difficile cells. The collected C. difficile cells were diluted to the same OD value (OD_600_ = 0.6) in 1 mL of DMEM medium. The obtained C. difficile cell solution was added to Caco-2 cells (2.58 × 10^6^ cells) at a multiplicity of infection (MOI) of 39 in a total volume of 2 mL of anaerobic DMEM (20% FBS). After a 40-min incubation, cells and bacteria were washed twice with 1 mL of reduced PBS, scraped, resuspended, serially diluted, and spread onto BHI agar plates (five plates for each dilution gradient). The adherent C. difficile strains were counted after 24 h ∼ 48 h of incubation. Experiments were carried out twice, and each was performed in triplicate (36).

### Scanning electron microscopy.

C. difficile cells were cultured in BHI medium to OD_600_ = 0.6 (prelogarithmic growth phase), and 5 mL cells were collected by centrifugation at 13,500 × *g* for 5 min. Bacteria cells were resuspended in 2.5% glutaraldehyde (dissolved in PBS buffer) and fixed overnight at 4°C. Then, cells are washed with PBS buffer (centrifuged at 9,500 × *g* for 5 min). Afterward, cells were washed three times with PBS buffer and then dehydrated in ethanol solutions of 50%, 70%, 90%, and 100% (vol/vol) for 5 min of each time. After serial dehydration, the cells were soaked in 50% ethanol 50% tert-butanol for 10 min, and then the soaking solution was changed to 100% tert-butanol. After 15 min, the samples were placed in a −20°C refrigerator to allow the tert-butanol to solidify. The solidified samples were dried in a vacuum-freeze dryer. The cells’ powder was carefully picked with a toothpick and sprayed with gold for scanning electron microscopy (SEM), and the cells were observed with a HITACHI S-3400 SEM (37).

### Autolysis assay.

Overnight cultures of C. difficile WT, Δ*cwp66* mutant, and ::*cwp66* strains were diluted to OD_600_ = 0.05 in BHI and incubated at 37°C until OD_600_ = 0.5. Bacterial cells were collected, washed twice, and resuspended in 50 mM potassium phosphate buffer (pH = 7.0), containing 0.01% Triton X-100, to OD_600_ = 0.5. The OD_600_ of the suspensions were then measured every 20 min at 37°C (OD_600_-M), the percentage of unautolysed cells was calculated as (OD_600_-M/0.5) * 100% (38). The untreated cells in the BHIS medium with the same inoculation ratio were set as the control group.

### pH tolerance assay.

The C. difficile WT, Δ*cwp66* and ::*cwp66* strains were cultured to OD_600_ = 0.5, 1 mL cells were centrifuged and resuspended in 0.5 mL of BHIS medium, which was adjusted to different pH value (pH = 1, 2, 3, 4, 5, 6, 7, 8, 9, 10, 11, 12). Cells were incubated in the BHI medium of different pH for 1 h. Afterward, 1 μL of the culture was dotted on BHI solid plate with pH = 6.8 and incubated in an anaerobic chamber for 24 h ∼ 48 h, and the growth of the cells was observed (39).

### Growth and cell motility assay.

The C. difficile strains were inoculated in the BHIS medium with 1% (vol/vol) inoculum. Inoculated tubes were incubated at 37°C in an anaerobic chamber without shaking. The OD_600_ values were measured at 6 h, 12 h, 24 h, 36 h, 48 h, and 72 h, and three biological repeats were carried out for each strain. The C. difficile strains were punctured with a sterilized inoculation needle in BHI semi-solid medium containing 0.5% agar powder (W/V). After incubation overnight at 37°C anaerobically, cell motility was recorded (40).

### Tolerance of C. difficile strains to hydrogen peroxide.

In each well of a 96-well plate, 200 μL of BHIS medium was supplemented with hydrogen peroxide (H_2_O_2_) at final concentrations of 1, 000 nM to 0 nM (decrease 25 nM sequentially). The overnight culture of C. difficile WT, Δ*cwp66*, and ::*cwp66* strains were inoculated into the H_2_O_2_-supplemented BHI medium with an inoculum of 5%. The absorbance value at OD_600_ was measured using a spectrophotometer (BioTeck Synergy 2) after being incubated anaerobically at 37°C for 24 h (41).

### RNA sequencing.

Total RNA was isolated using the TRIzol Reagent (Invitrogen Life Technologies). Quality and integrity were determined using a NanoDrop spectrophotometer (Thermo Scientific) and a Bioanalyzer 2100 system (Agilent). For mRNA sequencing, Ribo-Zero rRNA Removal Kit was used (Illumina, San Diego, CA, USA). Random oligonucleotides and SuperScript III were used to synthesize the first-strand cDNA. Second-strand cDNA synthesis was performed using DNA polymerase I and RNase H. Remaining overhangs were converted into blunt ends via exonuclease/polymerase activities, and the enzymes were removed. After adenylation of the 3′ ends of the DNA fragments, Illumina PE adapter oligonucleotides were ligated to prepare for hybridization. To select cDNA fragments of the preferred 300 bp in length, the library fragments were purified using the AMPure XP system (Beckman Coulter, Beverly, CA, USA). DNA fragments with ligated adaptor molecules on both ends were selectively enriched using Illumina PCR Primer Cocktail in a 15-cycle PCR. Products were purified (AMPure XP system) and quantified using the Agilent high sensitivity DNA assay on a Bioanalyzer 2100 system (Agilent). The sequencing library was then sequenced on a NextSeq 500 platform (Illumina) by Shanghai Personal Biotechnology Co. Ltd (42).

### Transcriptome analysis flow.

The sequencing data were analyzed as follows: (i) Quality control: samples are sequenced on the platform to get image files, which are transformed by the software of the sequencing platform, and the original data in FASTQ format (Raw Data) is generated. Sequencing data contains several connectors, low-quality Reads, so we use Cutadapt (v1.15) software to filter the sequencing data to get high-quality sequences (Clean Data) for further analysis (ArrayExpress accession E-MTAB-11180, https://www.ebi.ac.uk/). (ii) Reads mapping: the reference genome and gene annotation files were downloaded from the genome website. The filtered reads were mapped to the reference genome using HISAT2 v2.0.5. (iii) Differential expression analysis: we used HTSeq (0.9.1) statistics to compare the read count values on each gene as the original expression of the gene and then used FPKM to standardize the expression. Then differential expression of genes was analyzed by DESeq (1.30.0) with screened conditions as follows: expression difference multiple |log_2_FoldChange| > 1, significant *P*-value < 0.05. At the same time, we used the R language Pheatmap (1.0.8) software package to perform bi-directional clustering analysis of all different genes of samples. We used the heatmap according to the expression level of the same gene in different samples and the expression patterns of different genes in the same sample with the Euclidean method to calculate the distance and the Complete Linkage method to cluster (4).

The GO and KEGG enrichment analyses: we mapped all the genes to Terms in the Gene Ontology database and calculated the numbers of differentially enriched genes in each term. Using topGO to perform GO enrichment analysis on the differential genes, calculate *P*-value by hypergeometric distribution method (the standard of significant enrichment is *P*-value <0.05), and find the GO term with significantly enriched differential genes to determine the main biological functions performed by differential genes. ClusterProfiler (3.4.4) software was used to carry out the enrichment analysis of the KEGG pathway of differential genes, focused on the significant enrichment pathway with *P*-value <0.05.

### Statistical methods.

Prism 8 (Version 8.2.1) and R software (version 4.1.0) were used for statistical analysis. Student’s *t* test was used to compare the differences between groups, and the results were expressed as mean ± standard deviation, with a test level of α = 0.05, and *P* < 0.05 was statistically significant (*n* = 3). In Fig. 4, n, *P* > 0.1; ., *P* > 0.05, *, *P* < 0.05; **, *P* < 0.01; ***, *P* < 0.001.

### Data availability.

The RNA-seq raw data (RNA-seq of Clostridioides difficile Δ*cwp66* mutant against wild-type control) was deposited in the ArrayExpress database (https://www.ebi.ac.uk/fg/annotare/login/) under the accession number of E-MTAB-11180.
