# Supplementary material for: The cwp66 Gene Affects Cell Adhesion, Stress Tolerance, and Antibiotic Resistance in Clostridioides difficile
Source: Microbiol Spectr. 2022 Mar 31;10(2):e02704-21. doi: 10.1128/spectrum.02704-21 (PMC9045246; doi:10.1128/spectrum.02704-21)
Supplement: SUPPLEMENTAL FILE 1 — Supplemental material. Download SPECTRUM02704-21_Supp_1_seq13.pdf, PDF file, 0.9 MB [file spectrum02704-21_supp_1_seq13.pdf]

Supplemental materials

Supplemental Figures

Figure S1 Changes of resistance to antibiotics of the Δ*cwp66* mutant

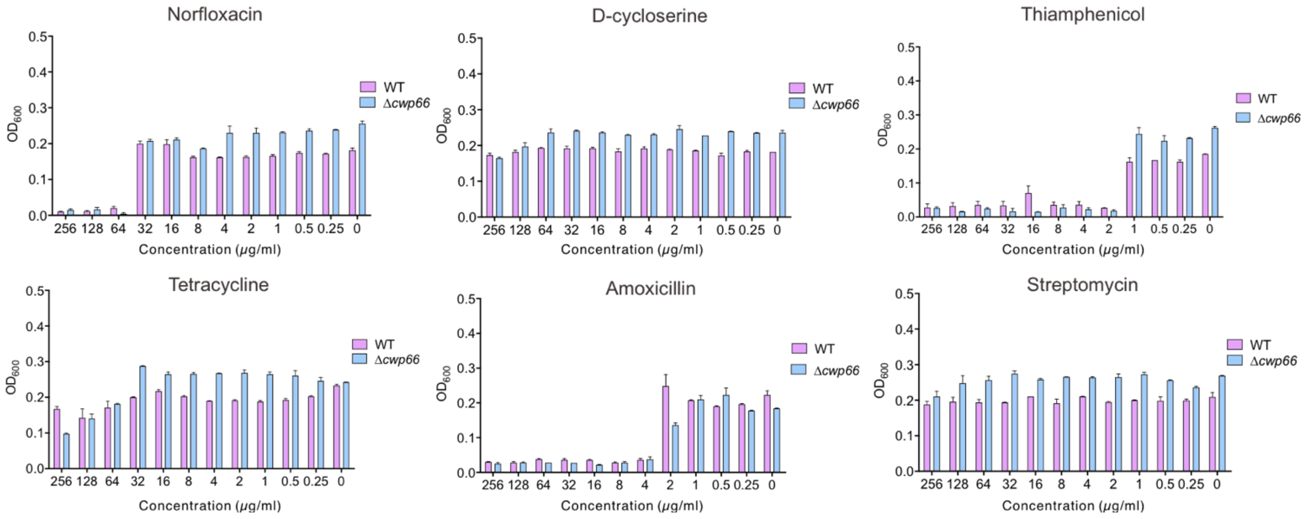

Figure S2 Gene sequence and toxins production level of Δ*cwp66* mutant

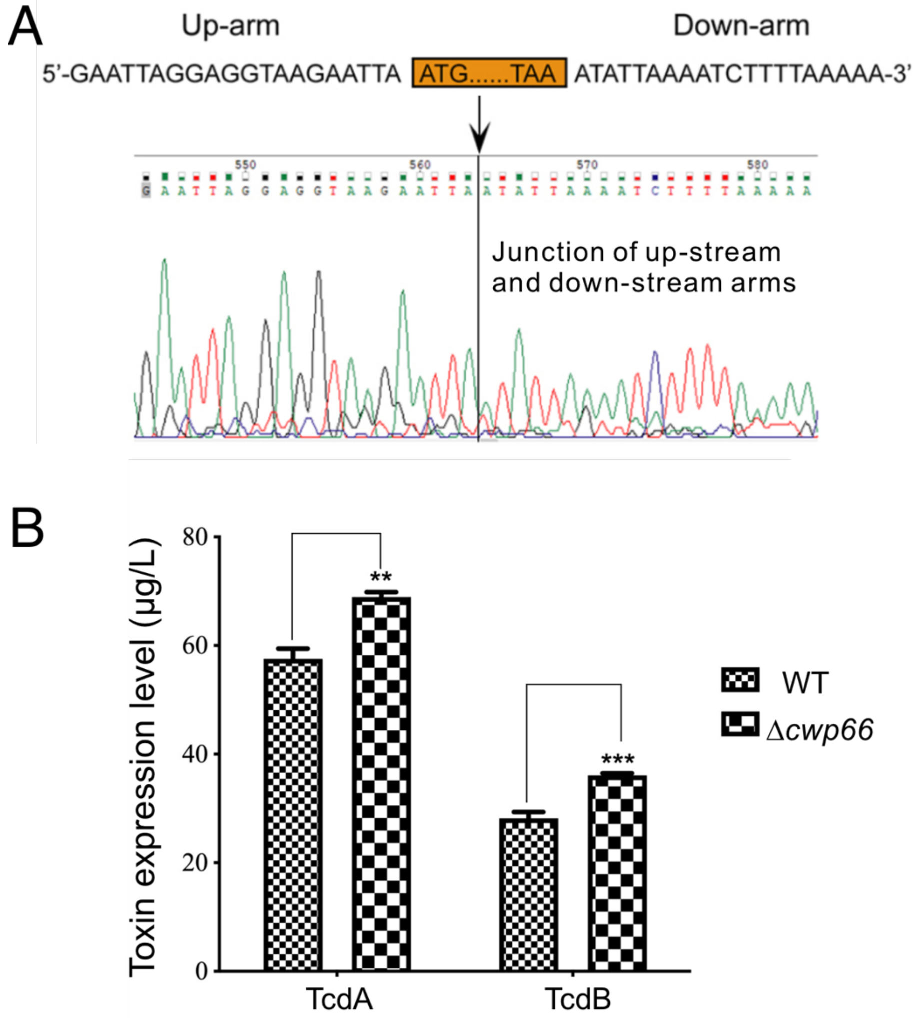

Figure S3 Sole carbon source utilization of different strains

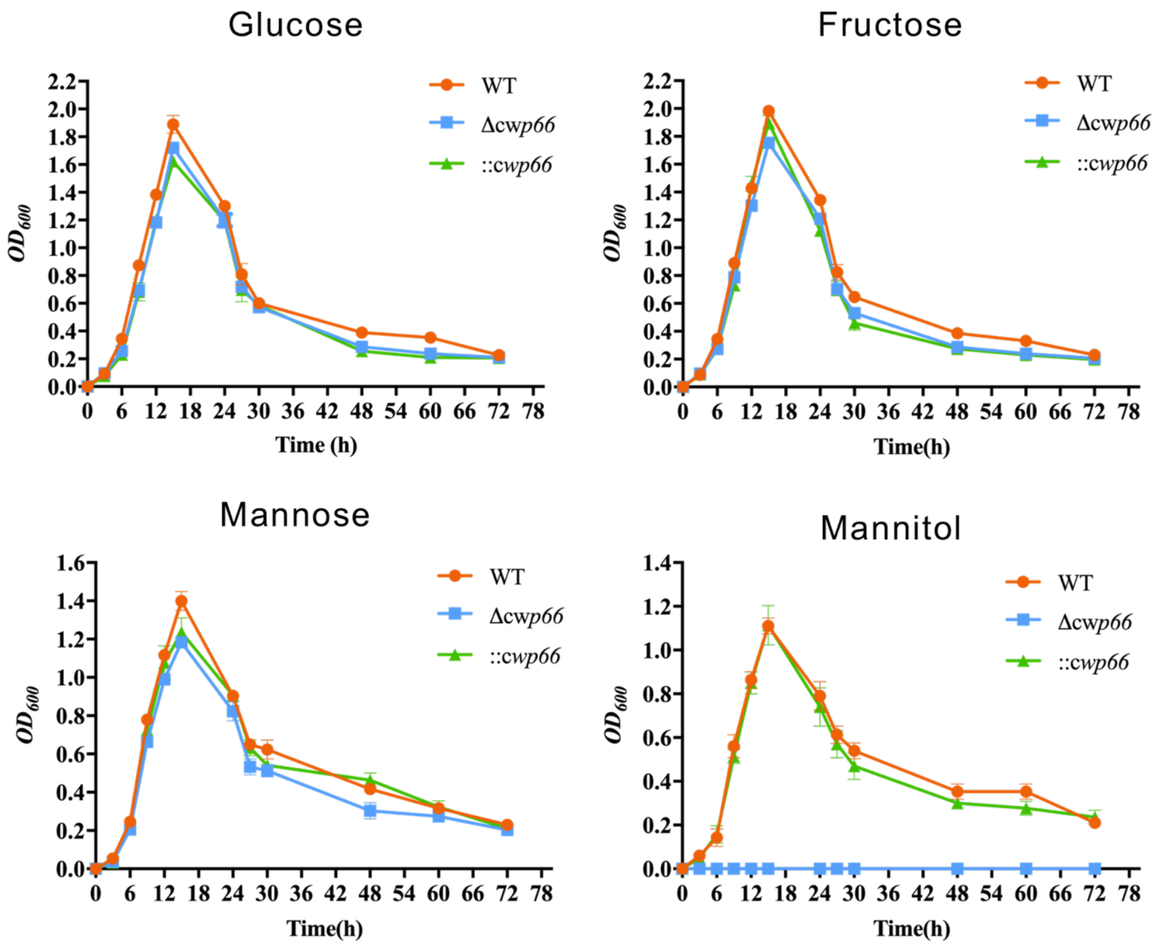

Supplemental legends

Figure S1 Changes of resistance to antibiotics of the Δ*cwp66* mutant

The vertical coordinate is the value of OD_{600}, and the horizontal coordinate is the antibiotic concentration (μg/mL). The blue and magenta bar indicates the OD_{600} values of Δ*cwp66* mutant and WT strain at different antibiotic concentrations. Compared with the WT strain, the Δ*cwp66* mutant strain showed similar MIC to norfloxacin, D-cycloserine, thiamphenicol, tetracycline, amoxicillin, and streptomycin.

Figure S2 Gene sequence and toxins production level of Δ*cwp66* mutant

(A) Verification of Δ*cwp66* mutant by using gene sequencing. The orange box represents the ORF of the *cwp66* gene, and the black arrow indicates the junction of up-stream and down-stream arms. The *cwp66* gene knockout resulted in the 3' end of the upstream homologous arm being linked to the 5' end of the downstream homologous arm, and DNA sequencing results confirmed this linkage; (B) The toxin concentrations of WT and Δ*cwp66* strains in the culture supernatant were measured, and the Δ*cwp66* mutant released more toxins in the culture supernatant than that of the WT strain. Student *t-test* was used to compare the differences between groups, and the results were expressed as mean ± standard deviation, with a test level of $\alpha = 0.05$. The '**' represents $P < 0.01$ and '***' represents $P < 0.001$, compared with the WT group.

Figure S3 Sole carbon source utilization of different strains

The vertical coordinate is the value of OD_{600}, and the horizontal coordinate is the incubation time (h). Growth curve of the WT, Δ*cwp66* and ::*cwp66* mutants in the CDMM medium supplemented with glucose (A), fructose (B), mannose (C), or mannitol (D) as the sole carbon source. Compared to the WT strain, the Δ*cwp66* mutant lost the ability to utilize mannitol, whereas the mannitol utilization-defective phenotype was rescued in the ::*cwp66* mutant.

Supplemental Tables

Table S1 Bacterial strains and plasmids used in this study.

Strains or Plasmids	Relevant characteristics	Reference or source
**Strains**		
E. coli		
NEB Express Competent *E. coli* (High efficiency)	*fhuA2 [Ion] ompT gal sulA11 R(mcr-73::miniTn10--Tet^S)2[dcm]R(zgb-210::Tn10--Tet^S)endA delta(mcrC-mrr)114::IS10*	NEB
E. coli CA434	*hsd20(r^B-, m^B-), recA13, rpsL20, leu, proA2,* with IncPb conjugative plasmid R702	(1)
Clostridium difficile		
C. difficile 630	WT stain	From Dr. Yi Wang (Auburn University)
Δ*cwp66*	Derived from *C. difficile* 630, Δ*cwp66*	This work
Δ*PaLoc*	Derived from *C. difficile* 630, Δ*PaLoc*	(2)
::*cwp66*	Derived from Δ*cwp66*, over expression of *cwp66* gene in Δ*cwp66* mutant.	This work
**Plasmids**		
pMTL83151	pBP1 ori, Cm^R, ColE1 ori, TraJ, *lacZ* α fragment	(3)
pWH34	Derived from pMTL82151, *E. coli-C. difficile* shuttle vector, iLacP::AsCpf1, *BtgZ*I-*BtgZ*I double sites, "Chassis" plasmid for gene-targeting plasmid construction, Cm^R/Tm^R	(4)
pWH37	Derived from pWH34, iLacP::AsCpf1, sRNAP::crRNA (23-nt repeat), Cm^R/Tm^R, PCR template for generating sRNAP::crRNA for retargeting	(4)
pWH55	Derived from pWH34, targeting cell wall protein *cwp66* (gene, Cm^R/Tm^R	(4)
pZQS1	Derived from pTML83151, iLacP::*cwp66*	This work

Table S2 Primers used in this study.

Primers	Sequences (5'-3')
**pWH55**	
YW3304	ATTATTAGCTGCTAATACACCCACTGCATCTACAAGAGTAGAAATTAATGGTGGAATGATAAGGGTT
YW3305	AGATGCAGTGGGTGTATTAGCAGCTAATAATTTCTACTCTTGTAGATTAGCTGTGAAGCAATGGGTGTAAAA
YW3306	TTTTGTTTTTAAAAGATTTTAATATTAATTCTTACCTCCTAATTCTTTCG
YW3307	CGAAAGAATTAGGAGGTAAGAATTAATATTAAAATCTTTTAAAAACAAAA
YW3308	CTCCATGGACGCGTGACGTCGACTCGCTGAGACAGGTATTGTTGACACTA
YW2369	TGGAGGGTCATGTTCTTATC
YW2370	GCTGAGACAGGTATTGTTGACA
**pZQS1**	
HW544	attcgagctcggtacccgggTTATATACTTGGTTTATTTACTTGATTAT
HW545	caaagacactatctttttgatattttcatTTCAGCCCTCCTGTGAAATT
HW546	AATTTCACAGGAGGGCTGAAatgaaaatatcaaaaagatagtgtctttg
HW547	gacgcgtgacgtcgactctagagTtaaaatccatcatctgtagcgttag

Supplementary References

1. Williams DR, Young DI, Young M. 1990. Conjugative plasmid transfer from *Escherichia coli to Clostridium acetobutylicum*. Journal of General Microbiology 136:819–826.

2. F.Q. Rao, Y.M. Cheng, C.X. Wu, Y. Wang, G.Z. Cui, X.L. Qi, W. Hong. 2019. Knockout of PaLoc Toxicity Loci to Construct Non-toxic *C. difficile* Strain. Journal of Guizhou Medical University 1128–1133.

3. Heap JT, Pennington OJ, Cartman ST, Minton NP. 2009. A modular system for *Clostridium* shuttle plasmids. Journal of Microbiological Methods 78:79–85.

4. Hong W, Zhang J, Cui G, Wang L, Wang Y. 2018. Multiplexed CRISPR-Cpf1-Mediated Genome Editing in *Clostridium difficile* toward the Understanding of Pathogenesis of *C. difficile* Infection. ACS Synth Biol 7:1588–1600.
